# Marginal Eyespots on Butterfly Wings Deflect Bird Attacks Under Low Light Intensities with UV Wavelengths

**DOI:** 10.1371/journal.pone.0010798

**Published:** 2010-05-24

**Authors:** Martin Olofsson, Adrian Vallin, Sven Jakobsson, Christer Wiklund

**Affiliations:** Department of Zoology, Stockholm University, Stockholm, Sweden; University of Oxford, United Kingdom

## Abstract

**Background:**

Predators preferentially attack vital body parts to avoid prey escape. Consequently, prey adaptations that make predators attack less crucial body parts are expected to evolve. Marginal eyespots on butterfly wings have long been thought to have this deflective, but hitherto undemonstrated function.

**Methodology/Principal Findings:**

Here we report that a butterfly, *Lopinga achine*, with broad-spectrum reflective white scales in its marginal eyespot pupils deceives a generalist avian predator, the blue tit, to attack the marginal eyespots, but only under particular conditions—in our experiments, low light intensities with a prominent UV component. Under high light intensity conditions with a similar UV component, and at low light intensities without UV, blue tits directed attacks towards the butterfly head.

**Conclusions/Significance:**

In nature, birds typically forage intensively at early dawn, when the light environment shifts to shorter wavelengths, and the contrast between the eyespot pupils and the background increases. Among butterflies, deflecting attacks is likely to be particularly important at dawn when low ambient temperatures make escape by flight impossible, and when insectivorous birds typically initiate another day's search for food. Our finding that the deflective function of eyespots is highly dependent on the ambient light environment helps explain why previous attempts have provided little support for the deflective role of marginal eyespots, and we hypothesize that the mechanism that we have discovered in our experiments in a laboratory setting may function also in nature when birds forage on resting butterflies under low light intensities.

## Introduction

Eyespots on the peripheral parts of butterfly wings have interested scientists for centuries and are considered to be part of the ground plan of the largest butterfly family, the Nymphalidae [1]. Several recent studies have increased our understanding of the genetic background for the evolution and development of butterfly eyespots [2]–[4]. In contrast, the adaptive significance of most eyespots is still poorly understood. Eyespots have traditionally been thought to have one of two distinctly different functions, either to intimidate predators making them abandon their attacks or to deflect attacks to non-vital body parts such as the wing margins [5]–[7]. There is now convincing evidence that large, dorsal eyespots can effectively intimidate small bird predators [8]–[11] but recent attempts to demonstrate the deflective role of smaller marginal eyespots have provided no support for this function [6], [12], [13].

Most predators target their prey by attacking essential body parts to avoid prey escape and to reduce other costs associated with attacks that are not instantly lethal [5], [14]. Prey adaptations, such as marginal eyespots, that encourage predators to attack less vulnerable body parts are hence expected to evolve [5], [15]. Birds are major predators of butterflies and it is likely that adaptive coloration and patterns on butterfly wings are targeted on avian predators and hence attuned to deceiving the sensory systems of birds [16]–[18]. Most bird species are sensitive to short-wavelength light [19]–[21] and light in the ultraviolet range (300–400 nm) appears to attract the attention of foraging birds [22]–[25]. Insectivorous birds characteristically forage not only during daytime but also under lower light intensities, especially at the break of dawn [26], [27]. Light reaching the earth around sunrise and sunset is typically predominated by shorter wavelengths (<450 nm) [28] and this shift towards shorter wavelengths is known to increase the brightness contrast between a white coloration, such as that of the pupil of the marginal eyespots, and the background [29]. Among butterflies, deflecting predator attacks is likely to be particularly important at dawn when low ambient temperatures make escape by flight impossible.

**Figure 1:**
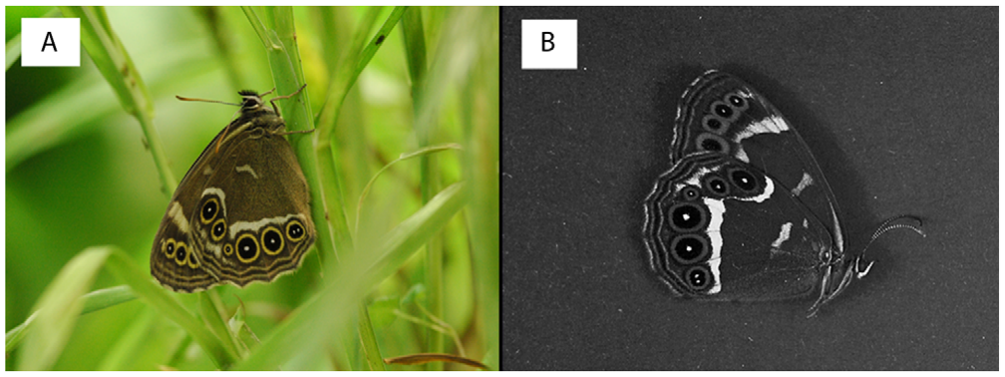
Photography's of the prey, the woodland brown butterfly, *Lopinga achine*. **a**. The woodland brown butterfly, *L. achine*, photographed in natural light. **b**. The butterfly photographed with a UV filter (Schneider 49 ES Ultraviolet Black 403 which passes UV A radiation (320 to 385 nm)) revealing the strongly UV-reflecting pupils on the hindwing eyespots.

Marginal eyespots are nearly ubiquitous in the butterfly subfamily Satyrinae, and each eyespot is composed characteristically of a central broad-spectrum reflective white pupil, which in turn is concentrically surrounded by a wider black ring and a narrower yellowish ring, altogether forming a highly contrasting wing pattern [2]–[4] (Fig. 1a and 1b). These marginal eyespots are usually more accentuated on the ventral surface of the butterfly wings, which is exposed when the butterfly is resting. It has been shown that the eyespots on the forewing of the satyrine butterfly, *Bicyclus anynana,* are subject to sexual selection [30] and, more recently, that their function is linked to the UV-reflecting white pupil on the dorsal surface of the forewings [31], [32]. However, there is no evidence that the ventral marginal eyespots have any influence on female mate choice and Robertson & Monteiro [32] instead suggested an anti-predatory function of such eyespots. These eyespots typically show seasonal variation, especially in tropical environments, and are much larger and more numerous in the wet season form than in the dry season form [6], [12], [33]. Differences in butterfly activity and predator composition between the seasons are thought to explain the greater need for crypsis in the dry season when butterflies can spend an extended time period in diapause without moving from their resting place, compared to a defence incorporating the use of deflective marginal eyespots in the rainy season when the butterflies are more active and expose themselves more openly to predators [33]. There is strong evidence that the eyespots in the wet season form make the butterflies less camouflaged if they were to appear in the brownish surroundings which are prominent during the dry season [13] but the adaptive (anti-predator) function of eyespots in the wet season form in the wet season surroundings still needs more empirical support. Lyytinen and colleagues [13] showed that naïve pied flycatchers “caught and ate fewer spotted butterflies than did adult birds”. This result is interesting and suggests that predation from juvenile birds may be instrumental in understanding wing pattern polyphenism in *B. anynana*. However, the mechanistic reason for this finding needs further study, because analysis of wing damages on the two attacked butterfly forms did not reveal any evidence that the eyespots had influenced how the birds had initially targeted their butterfly prey. Furthermore, their experiments showed no clear distributional distinctions of the initial strikes (which should be expected if marginal eyespot are to deceive predators) on the two seasonal forms of *B. anynana*, neither from experienced birds nor from reptile predators [6], [12], [13]. Hence, evidence strongly indicates that eyespots do not generally misdirect predator attacks, but might be effective only under certain light conditions [12] such as at dawn, dusk or in habitats where light conditions are poor.

In tropical environments, most butterflies with eyespots inhabit the shady undergrowth and the forest floor and are less frequently found in the more sun-exposed habitat above the dense foliage [34]. Although the stratification of butterfly species cannot safely be separated from phylogenetic relatedness, this vertical habitat stratification is consistent with the idea that light conditions play a role for the evolution and maintenance of eyespots in butterflies. It is conceivable that the strong reflectance of the pupil of the eyespot is important, especially because the composition of light at dawn and dusk is particularly rich in short wavelengths which increase the brightness contrast between the eyespot pupils and the background. Moreover, the reflection of the eyespot pupils extends into the ultraviolet region, and birds are known to use UV-reflection as a signal for discovering their prey [22], [24]. Here, we test if the deflective capacity of marginal eyespots is influenced by the strong broad-spectrum reflective properties of the white eyespot pupils, and whether ambient light conditions influence how birds aim their attacks; we do this by staging experiments between a generalist bird predator, the blue tit, *Cyanistes caeruleus*, and a butterfly with large marginal eyespots, the woodland brown, *Lopinga achine* (Fig. 1a), under different light intensity conditions with or without a UV light component.

## Results

**Figure 2:**
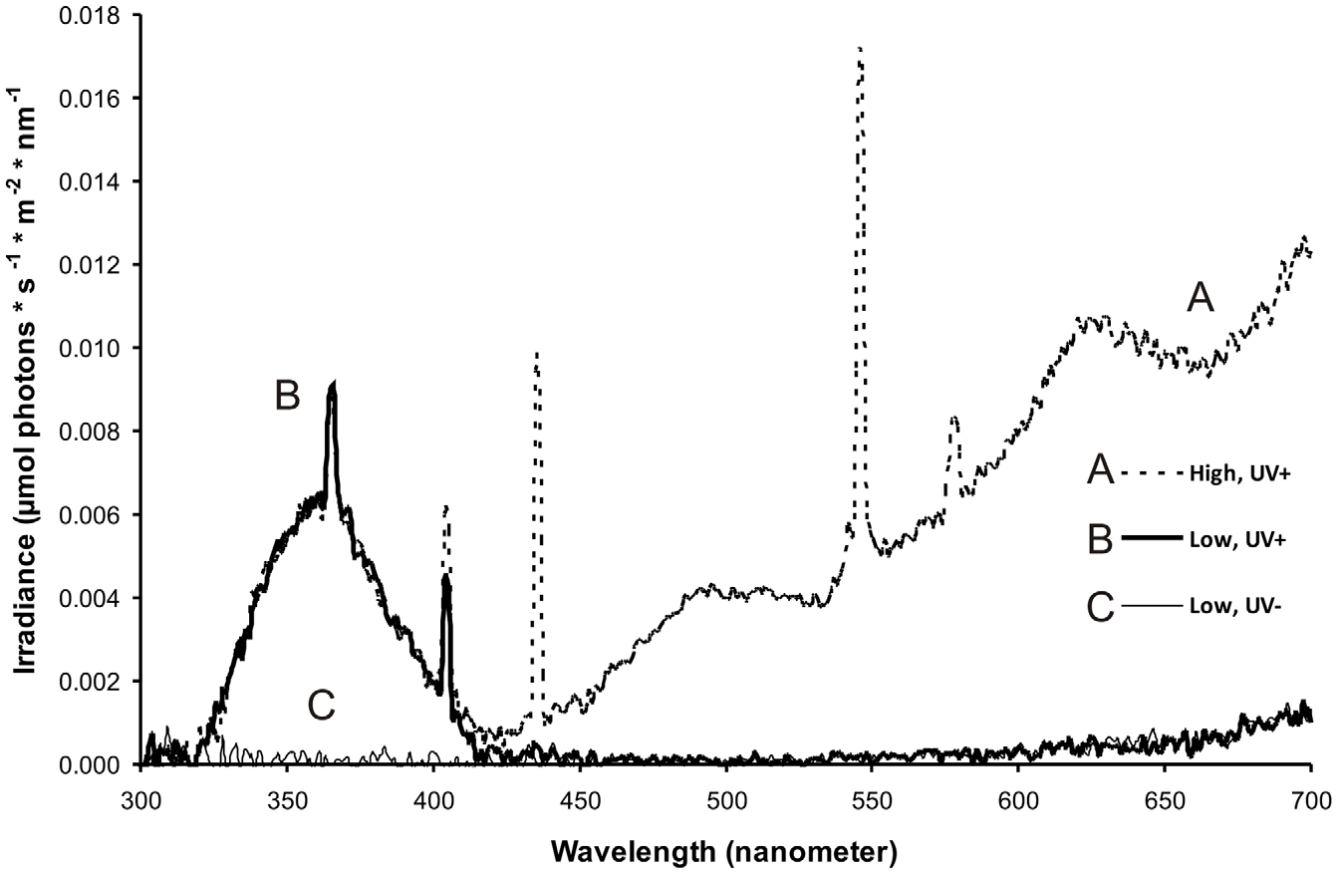
Light environments in the three treatments. Irradiance measurements in the experimental room demonstrate the difference in light composition between the three treatments (**A** High, UV+; **B** Low, UV+ and **C** Low, UV -).

**Figure 3:**
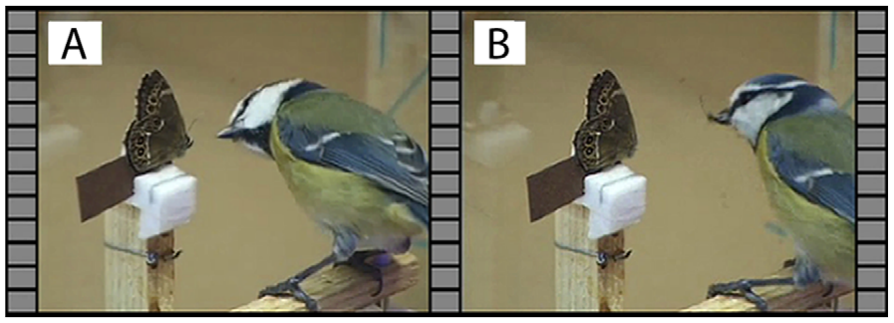
Bird attack on butterfly under high light intensity conditions with UV. **a**. Visual inspection of the butterfly by a blue tit–just preceding the bird's attack. **b**. The bird with the head of the butterfly in its beak after the attack.

**Figure 4:**
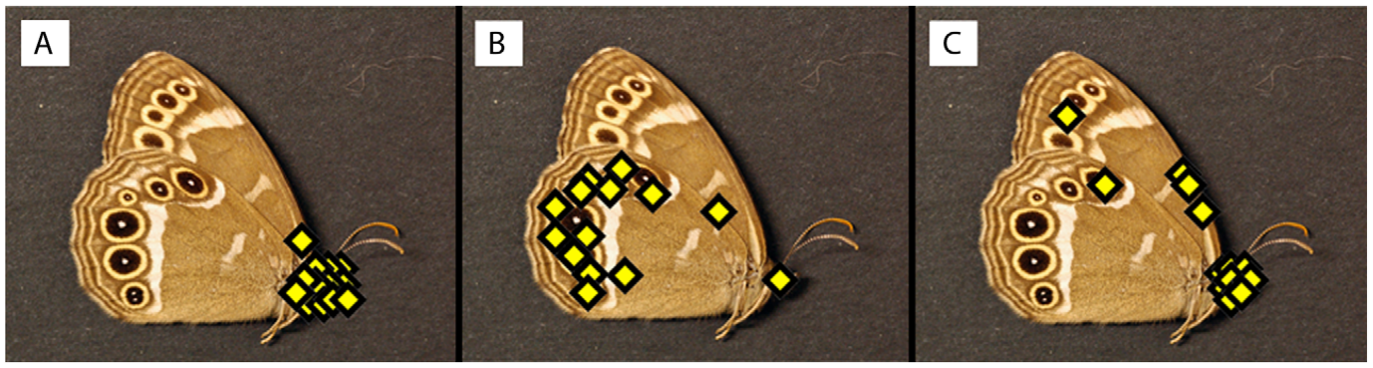
Distribution of attacks in the three treatments. **a**. Distribution of blue tit attacks under high light intensity in the presence of UV wavelengths (High, UV+, n = 13). **b**. Distribution of blue tit attacks under low light intensity in the presence of UV wavelengths (Low, UV+, n = 14). **c**. Distribution of blue tit attacks under low light intensity in the absence of UV wavelengths (Low, UV-, n = 13).

When we presented mounted butterflies in the high light intensity treatment with a UV component present (High, UV+, Fig. 2) all birds (n = 13) distinctly attacked the butterfly's head and decapitated the butterfly in one instant strike or seized it with the beak just behind the head (Fig. 3 and 4a); hence not a single attack was misdirected towards the eyespots.

When we reduced the light intensity with the UV-component intact (Low, UV+, Fig. 2), twelve birds (n = 14) misdirected their attacks and struck the marginal eyespots (Fig. 4b). In the twelve ‘eyespot attacks’ birds typically initiated the attack by quickly pecking towards the eyespots, whereupon six of the birds grasped the butterfly by its hindwing margins or by the upper part of the forewing and ripped wing pieces or the whole butterfly from its position; only two birds corrected their mistake and launched a second attack towards the head. The remaining six birds that attacked the eyespots either returned once more and pecked towards the eyespots or grasped the butterfly by its wings or flew away and never returned.

Interestingly, the deflective effect was drastically impaired also in the low light intensity treatment without a UV component (Low, UV-, Fig. 2), with only two birds (n = 13) misdirecting their attacks towards the marginal eyespots (Fig. 4c). The proportion of misdirected attacks was significantly larger in the Low, UV+ treatment compared to the Low, UV- and High, UV+ treatments; 86% of the birds attacked the eyespots at Low, UV+ but only 15% and 0% did so at Low, UV- and at High, UV+ (Fisher's Exact test, two-tailed: Low, UV+/Low, UV-, *p* = 0.00042; Low, UV+/High, UV+, *p*<0.0001).

## Discussion

We contend that our experiments are the first to demonstrate that natural marginal eyespots on a butterfly's wings can deflect predator attacks to these non-vital parts of the prey, and the highly context-dependent situation (low light intensities with enhanced UV-levels) in which the eyespots do so probably explains why previous experiments have not revealed that predators misdirect their attacks towards the eyespots [6], [12], [13]. Earlier studies have shown that artificial markings applied to peripheral body parts of prey, or models of prey, can influence how bird predators direct their attacks [15], [35], [36].

Recent experiments testing the deflective function of the eyespots in the butterfly *B. anynana* were performed under light conditions corresponding to daylight, but did not find that bird or reptile predators directed their attacks towards the wing margins more often on the wet season form bearing eyespots (or a mutant with greatly enlarge eyespots) compared to the dry season form with no or only vestiges of eyespots [6], [12], [13]. Under sunny conditions butterflies typically attempt to escape from attacking birds by simply flying away. However, as butterflies are ectothermic and dependent on warmth from the sun, escape by flight is unfeasible when ambient temperatures are low, and a butterfly when attacked at dawn can only escape by leaving its roosting place and dropping into the undergrowth [37]. Under such conditions escaping predation is likely to be increased if the initial attack by a bird is deflected to eyespots located on non-vital body parts. Evidence suggests that predation pressure is strong on resting butterflies [38], [39], which is the case during the early and light deficient hours at dawn when birds are resuming another day's search for food and butterflies are still resting on their night roost. This is likely to be important during the breeding season when naïve birds abound and evidence suggests that inexperienced birds play a role as selectors on butterfly eyespot patterning [13]. The extraordinarily strong deflective effect obtained in the low light intensity treatment with a UV component (Low, UV+) cannot be translated directly to natural conditions at dawn or dusk but suggests that the light conditions are instrumental for the deflective function of marginal eyespots in butterflies. The deflective effect may not be as strong under low light intensities in a natural setting as in our Low, UV+ treatment since the relative difference in intensity of short wavelengths (300–450 nm) compared to long wavelengths (450–700 nm) was somewhat more accentuated in our experiment compared to natural levels [28]. However, the absolute amount of short-wave light (<450 nm) in our Low, UV+ treatment is comparable to natural levels [28]. We contend that deflection is not an ‘all-or-nothing phenomenon’, and suggest that the deflective effect of butterfly eyespots probably increases with decreasing light levels at dusk, and conversely decreases with increasing light intensities at dawn. Moreover, the low light intensity conditions used in the Low (UV+ and UV-) treatments are not lower than light levels experienced by foraging birds in the field; we base this inference on our recording, by using a movement initiated IR-sensitive camera, Cuddeback Digital, how a great tit, *Parus major*, entered a house through a paneless window and caught a small tortoiseshell butterfly, *Aglais urticae*, that was hibernating on the wall in an unheated attic; the attack occurred at 10∶29 am on 9 November 2007. The light intensity at the time was indeed as low as in our low light intensity treatment without UV (Low, UV-) (∼15 lux, measured with a Delta Ohm Photo-Radiometer HD2302.0).

Hence, small passerines such as the great tit do forage actively and indeed manage to find insect prey under conditions when the light intensity is quite low, although it remains to be assessed to what extent this can help explain the general adaptive significance of marginal eyespots on butterfly wings under natural conditions. In addition, our experiments clearly show that birds search for food and attack butterflies at quite low light intensities and that, despite low light levels, birds were obviously able to see the whole butterfly, both in the Low, UV+ treatment as evidenced by the observation that that two birds aimed their initial attack towards the butterfly head, and two more birds attacking the butterfly head after an initial misdirected attack towards the marginal eyespots, and in the Low, UV- treatment as evidenced by the majority of birds directing their attacks towards the head of the butterfly. In our High, UV+ treatment all birds distinctly attacked the butterfly head or seized it just behind the head, suggesting that they would do so also in natural daylight.

It is customarily assumed that different kinds of deflective markings render prey more conspicuous (in this case, bird attraction to a broad-spectrum reflective white eyespot pupil that reflects light into ultraviolet wavelengths and which becomes increasingly conspicuous when the ambient light environment shifts to shorter wavelengths at dawn [29]) and hence more easily detected compared to cryptic prey without such markings [5], [40]. However, the cost of the added conspicuousness brought about by the marginal eyespots could be balanced if the increased probability of escape and survival after a misdirected attack is high enough to outweigh the cost of an increased risk of detection [40].

Although our experiments clearly show that the relative proportion of short and long wavelengths can influence how birds aim their attacks, further experiments will have to be performed in the field to test the ecological relevance of our discovery. Such experiments could make use of a movement initiated and light sensitive camera that can operate under low natural light conditions. Questions that seem worthwhile addressing would be (i) to determine whether birds target their prey differently dependent on the light environment and are more likely to attack marginal eyespots at dawn, dusk or in shady habitats, (ii) to what extent a butterfly that survives the first misdirected attack is able to survive after adopting its secondary defence and dropping into the undergrowth, and (iii) to what extent different birds use conspicuous patterns on the wings of butterflies that reflect light into ultraviolet wavelengths as a cue when foraging for insects. Marginal eyespots are common in many different groups of butterflies [4], and come in a variety of sizes and numbers even among closely related species suggesting that the adaptive significance can differ between species. Nonetheless, here we present novel evidence that marginal eyespots in butterflies can deflect predator attacks, and suggest a possible mechanism, where ambient light conditions are instrumental for the anti-predator function of such features.

## Materials and Methods

### Ethics Statement

Birds were captured with permission from the Swedish Bird Ringing Center (permission 613). Housing of birds and the experimental setup was reviewed and approved by the regional ethical committee (permit Linköpings djurförsöksetiska nämnd Dnr 2–07). Butterfly larvae were descendants from wild-caught females collected at Gotland, Sweden (permit Länstyrelsen Gotlands län, Dnr 522-1969-07).

The woodland brown butterfly *L. achine* was chosen as a suitable prey for testing the deflection hypothesis, because this satyrine has large marginal eyespots with broad-spectrum reflectance in their white eyespot pupils that strongly reflect light well into ultraviolet wavelengths (Fig. 1a and 1b). The butterflies used in the experiments had been reared as larvae on the grass *Dactylis glomerata*, and were put in a glassine envelope within an hour after emergence and euthanized by freezing at 25°C below zero. Before being presented to the birds the butterflies were mounted on a 1×4 cm piece of brown paper with superglue exposing the right ventral surface of the wings.

The experiments were carried out at Tovetorp Zoological Research Station, located in the southeast of Sweden, between March-April 2007 and February-March 2008. Blue tits, caught in trap-cages and in mist-nets at the research station, were chosen as predators. All birds in the study were experienced and had spent at least on summer out in the wild catching insects. Moreover, blue tits have four retinal cones of which one detects ultraviolet wavelengths [19], [21], [22]. The birds were housed indoors individually in cages (80×60×40 cm) and had access to water, sunflower seeds and tallow balls *ad libitum*. In addition, the diet was once a day supplemented with mealworms, *Tenebrio molitor*. The indoor lighting regime was set to correspond to that of the prevailing season. A total of 40 birds were used in the study (High, UV+, n = 13; Low, UV+, n = 14 and Low, UV-, n = 13).

All trials were performed in a room measuring 2.3×2.4×1.9 m with two walls supplemented with one-way windows for visual observations. The room was illuminated by four fluorescent tubes (Philips TL-D 90 Graphica Pro 36W/950) and four spotlights (Philips Daylight 60W/230V), which were mounted on the ceiling and covered with glass blocking shortwave light frequencies. The spotlights were connected to a light dimmer for light intensity adjustments. Ultraviolet light was provided by two standing spotlights (Fluorescent Lamp, SANEX STD-15W) at a 60 cm distance from the mounted butterfly. The experimental setup was composed of an 80×30 cm willow log (*Salix caprea*) and a 56×11.5 cm plank blocked up on the same level as one of the willow log ends. The plank had three small perches, gradually increasing in height. In front of the last perch the mounted butterfly was presented glued onto a slender piece of wood, and typically the blue tit would sit on its perch for a second or two before launching its attack (Fig. 3). To avoid bird attacks from any other direction than the intended a sheet of acrylic glass was erected behind the butterfly. A 1.6 m perch was placed at the other end of the log. Birds had fresh water accessible throughout the trials.

Before a trial began, all birds underwent an initial training procedure under high light intensity conditions with the mounted butterfly hidden from view by a piece of black paper. Two mealworms (*Tenebrio molitor*) were offered, one on the willow log and one immediately in front of the hidden butterfly. As soon as both mealworms were consumed we turned all lights off and removed the black paper that hid the butterfly from view via an attached nylon line from outside the room after which the mounted butterfly became fully visible just in front of the perch on which the bird had been sitting on when taking the second mealworm. A trial started when we lit the room in one out of three ways, either by only using dimmed light from the four spotlights (i) with UV spotlights turned on (Low, UV+) or (ii) with UV spotlights turned off (Low, UV-), or (iii) by using all light sources at their maximum effect (High, UV+). The light environment differed between the three treatments (Fig. 2); wavelengths between 320 and 420 nm were only apparent when the UV spotlights were turned on (High, UV+; Low, UV+), and these spotlights also provided one peak at about 410 nm. The light intensity was higher between 420 and 700 nm when all the light sources were turned on (High, UV+) compared to the two low light intensity treatments (Low, UV+; Low, UV-). Comparing the spectra in the light environment when the UV-spotlights were turned on (Low, UV+, High, UV+) with the normalised absorptance of the ultraviolet sensitive cone type of the blue tit [21] shows that they are closely congruent and both peak around 360–370 nm. Irradiance was measured with a spectrophotometer (Avaspec-2048-USB2-UA) equipped with a cosine corrector. All trials were recorded with a digital video camera (Sony DCR-VX1000E) placed inside the experimental room and focused on the mounted butterfly, allowing us to analyse exactly towards what body parts the birds directed their attacks.

After a completed trial, birds were transferred to their home cages and were allowed to rest and eat before being ringed and released back to the wild. All birds were healthy upon release and had to spend no more than one week in captivity and no individual bird or butterfly was used in more than one trial. The statistical analysis was performed in R 2.7.2 (R Development Core Team 2008).
